# PERIPHERAL BLOOD CELL MITOCHONDRIAL FUNCTIONS ARE ASSOCIATED WITH COGNITIVE PERFORMANCE AND ALZHEIMER’S DISEASE

**DOI:** 10.1093/geroni/igae098.0807

**Published:** 2024-12-31

**Authors:** Gargi Mahapatra, Jaclyn Bergstrom, Suzanne Craft, Anthony Molina

**Affiliations:** University of California San Diego, La Jolla, California, United States; UC San Diego, La Jolla, California, United States; Wake Forest University School of Medicine, Winston-Salem, North Carolina, United States; University of California San Diego, La Jolla, California, United States

## Abstract

Alzheimer’s disease (AD) dementia associates with systemic mitochondrial bioenergetic decline. We previously showed blood cell mitochondrial bioenergetics recapitulates bioenergetic capacity of highly metabolically active organs like brain, liver, and skeletal muscles, and peripheral blood mononuclear cell (PBMC) mitochondrial function relates to brain morphology. More recently, we examined PBMC and platelet bioenergetics in a cohort of older adults with dementia due to AD. We assessed mitochondrial function using complementary respirometric approaches in intact and permeabilized PBMCs and platelets from individuals with normal cognition (NC), mild cognitive impairment (MCI), and dementia due to probable AD (DEM). Cognitive abilities were assessed using Modified Preclinical Alzheimer’s Cognitive Composite (mPACC5) scores, and brain morphology using MRI. Our results indicate blood cells exhibit lower bioenergetic capacity associated with cognitive decline in MCI and DEM, with lowest capacities in DEM. Specifically, glucose-mediated respiration was significantly lower in DEM compared to NC. PBMC fatty-acid oxidation (FAO)-mediated respiration were progressively lower with MCI and DEM compared to NC, while platelet FAO-mediated respiration exhibited higher maximal respiration in MCI than NC and DEM. PBMC and platelet respiration positively correlated with mPACC5 scores and hippocampal volume, and negatively correlated with white matter hyperintensities. These findings indicate mitochondrial bioenergetic differences associated with cognitive abilities are systemic and blood-based bioenergetic profiling can be used as a minimally invasive approach for measuring mitochondrial alterations associated with dementia. Studies are underway to identify circulating factors underlying systemic bioenergetic decline and cellular mechanisms mediating mitochondrial dysfunction in PBMCs and platelets from older adults with cognitive impairment.

